# Physical Activity and Quality of Life After Distal Femur Tumor Resection and Limb Salvage

**DOI:** 10.3390/jfmk10010013

**Published:** 2024-12-31

**Authors:** Dimitra C. Galanis, Ioannis Zafeiris, Fotini M. Soukakou, Dimitra P. Papagelopoulos, Panayiotis Gavriil, Ioannis G. Trikoupis, Olga D. Savvidou, Konstantinos Vlasis, Vasileios A. Kontogeorgakos, Panayiotis J. Papagelopoulos

**Affiliations:** 1 First Department of Orthopedic Surgery, School of Medicine, National and Kapodistrian University of Athens, Attikon University General Hospital, 12462 Athens, Greece; dimikgalani@gmail.com (D.C.G.); zafeiris_ioannis@hotmail.com (I.Z.); fsoukakou@hotmail.com (F.M.S.); gavriilpan@gmail.com (P.G.); giannistrikoupis@gmail.com (I.G.T.); olgasavvidou@gmail.com (O.D.S.); vaskonto@gmail.com (V.A.K.); 2 School of Medicine, European University of Cyprus, Nicosia 2404, Cyprus; dimitrapapagelopoulos@gmail.com; 3 Department of Anatomy, School of Medicine, National and Kapodistrian University of Athens, 11527 Athens, Greece; kostasvlasis@gmail.com

**Keywords:** physical activity, sports activity, distal femur, bone tumors, sarcomas, resection, megaprosthesis, quality of life

## Abstract

**Background/Objectives**: Distal femur tumor resection with limb salvage is a demanding procedure that offers hope for patients by preserving the limb rather than opting for amputation. While limb salvage can improve both physical function and psychological well-being, there’s limited knowledge on how active patients remain afterward and how their Quality of Life (QoL) is affected, especially regarding physical activities and sports. This study investigates the quality of life of the patients through the development of motor activity, focusing on both physical and sporting activity of the above-mentioned individuals and their physical abilities to participate in activities of daily and sporting life after surgery. **Methods**: This study involved 16 patients aged 19–47 years who had undergone surgical resection and replacement of the distal femur for the treatment of sarcoma and were selected by random sampling from a total of 72 patients who had undergone a similar procedure. To explore the topic in depth, the researchers followed the triangulation method. From July 2023 to February 2024, we used surveys and interviews to explore their physical activity (PA) levels, sports participation, and QoL. This study included the International Physical Activity (PA) Questionnaire (IPAQ), the University of California and Los Angeles (UCLA) Activity Score, and semi-structured interviews. The data were analyzed using the statistical software packages SPSS 25 and Excel. **Results**: Most participants reported moderate to high levels of PA, according to IPAQ, and continued engaging in sports, with swimming, walking, and stair climbing being the most common activities. No significant relationship was found between their PA levels and factors such as age, BMI, or the side of the affected limb. Interviews showed that patients’ motivation and their surgeon’s guidance played key roles in their return to regular activities, though many exercised less frequently or intensely than recommended. **Conclusions**: Patients who undergo limb salvage surgery following distal femur tumor removal generally maintain a good level of physical activity, which supports their QoL. Encouraging these patients to stay active and even engage in sports appears feasible, especially when guided by medical professionals. These findings highlight the potential benefits of tailored rehab programs to improve long-term health and QoL in sarcoma survivors, although larger studies are needed for more comprehensive insights.

## 1. Introduction

Bone and soft tissue sarcomas comprise a rare heterogeneous group of malignant neoplasms, accounting for about 1% of all malignant tumors in adults [1], constituting up to 9% in young adults and 12% in the pediatric population.

Primary and metastatic bone tumors are often located at the distal femur and proximal tibia. Historically, these tumors were conventionally addressed either with arthrodesis or amputation [2]. Both operative techniques had many implications for limb function, as well as many psychological consequences for the patients and their overall well-being.

Research shows that currently 80–90% of patients with limb sarcomas avoid amputation, while survival rates and complete-healing rates are very encouraging. Overall, the current bibliography endorses limb-salvage surgical procedures, prioritizing patients’ safety in surgery and emphasizing the significance of preserving a functional limb [3].

Moreover, individuals undergoing knee tumor resection and limb salvage typically exhibit elevated self-perception in comparison to those undergoing amputation, whereas healthcare costs are higher for patients receiving amputation treatment. Similar survival rates were observed for both groups [4].

Regarding the choice of knee tumor resection and limb salvage for the treatment of sarcoma, there are many considerations, including bone and muscle tissue loss, reduction in muscle strength, impact on the survivor patients’ cardiorespiratory capacity and daily activities, and, in general, a negative effect on their quality of life. Patients with primary malignant bone tumors (e.g., osteosarcoma, chondrosarcoma, Ewing sarcoma) and soft tissue sarcomas (e.g., synovial sarcoma, liposarcoma) in lower limbs constitute a unique population that presents a likelihood significant reduction of physical activity (PA) following limb-salvage surgery or amputation [5]. The most commonly reported consequences of simple or complex surgical interventions for lower limb sarcomas in both pediatric and adult survivors include increased strain on walking and standing mechanisms, resulting in decreased speed and agility, lower limb weakness, restricted performance capabilities, and limitations in attending school or work [5,6,7]. Other studies have shown that patients treated for lower limb sarcomas experience severe symptoms, such as fatigue and anxiety [8].

In addition, significant emotional and physical fatigue, depression, anxiety, fear, diminished self-esteem, and alterations in self-perception are some of the side effects identified in cancer patients necessitating a prompt intervention in their lifestyle, specifically their diet and exercise program, but also their psychological state [9].

The above-mentioned studies show that undergoing knee tumor resection and limb salvage for sarcoma treatment impact various aspects of patients’ quality of life, including physical function, as well as emotional well-being, mental health, and social interactions.

Traditionally, physicians used to advise cancer patients to rest and abstain from Physical activity. However, studies carried out in the 1990s and 2000s questioned this guidance. In 2010, the American College of Sports Medicine concluded that PA is not only safe during cancer treatment but also post-treatment, potentially leading to enhanced health outcomes for patients [9]. Specifically, numerous studies have shown that PA enhances physical condition, physical function, and quality of life and alleviates cancer-related fatigue among various groups of cancer survivors [10].

According to the literature, survivor patients with musculoskeletal tumors are in the majority physically inactive [11]. While there is evidence that PA contributes positively to cancer survivors [5,12], there are no studies regarding the levels of sports activity in patients who have undergone tumor resection and reconstruction of discrete segments of the femur.

The purpose of the present study was to examine the kinetic profile of patients undergone knee tumor resection and limb salvage as part of sarcoma treatment, specifically focusing on their engagement in physical activities.

## 2. Materials and Methods

After obtaining Institutional Review Board approval, a study was conducted between July 2023 and February 2024. Sixteen patients with primary tumors of the distal femur who had undergone knee tumor resection and limb salvage for bone sarcoma treatment were recruited. The age range was from 19 to 47 years old. The patients were selected through random purposeful sampling from a pool of 72 patients who were diagnosed with primary tumor of the distal femur and underwent limb salvage surgery for sarcoma treatment at least three years prior.

Out of these 72 patients, 17 patients did not meet the age criterion, 8 patients did not meet the criterion of being three years post-surgery, and 2 patients were deceased. From the remaining 45 patients, 16 patients (n = 16) were selected using the random sampling method. A total of 12 of the 16 patients were males and 4 patients were females aged between 19 and 47 years old, with time elapsed since surgery ranging from 3 to 24 years.

From the patients of our sample, 9 patients were diagnosed with osteosarcoma, 4 with chondrosarcoma, 2 of them with Ewing sarcoma, and 1 with pleomorphic sarcoma of the distal part of the femur.

Concerning the affected limb, the right lower limb was affected for 4 patients, while the left lower limb was affected in the remaining 12 patients (Table 1).

Moreover, regarding the surgical procedure of knee tumor resection and limb salvage, an average bone resection of 16 cm (ranging from 12 cm to 25 cm) was performed, along with the removal of muscles for the quadriceps group, including vastus intermedius, vastus medialis, or vastus lateralis, or both muscles depending on the tumor location. However, the rectus femoris muscle was preserved in all cases. During surgery, the remaining muscles were reconstructed using either the sartorius muscle, the adductor muscles, or, in some cases, the gastrocnemius muscle. In most cases, Trevira fabric, a synthetic material, was used for the attachment of soft tissues. The surgeries were performed at Attikon University General Hospital, First Department of Orthopaedic Surgery, Athens University Medical School, by the same surgeon (PJP) and surgical team.

For the purpose of data collection, two questionnaires were selected, and a semi-structured interview was designed to record physical activity. These tools were chosen from pre-existing protocols based on the following criteria: their alignment with the study’s objectives, frequency of use in similar studies, and the reliability and validity of the questionnaires. Specifically, we selected the following tools:

(a) International Physical Activity Questionnaires (IPAQs sort self-answered 8 items—in Greek) [13,14];
(b) UCLA Activity Score test, University of California, Los Angeles [15,16];
(c) Semi-structured interview (reviewed and approved by the Institutional Review Board: ‘Attikon’ University General Hospital, Athens, Greece, ΕΒΔ A3/10-05-2023)

Data for the questionnaire completion were collected via email, while the semi-structured interviews were conducted over the phone or through platforms chosen by each patient, such as Skype, Messenger, WhatsApp, or Viber.

The IPAQ and the UCLA Activity Score test were analyzed according to the instructions of the creators [15].

The analysis of the semi-structured interview data was conducted through content analysis, following the stages proposed by Berelson [17]. The responses were coded by two coders and were based on the research questions in a transparent and well-justified manner [18]. The percentage of agreement between the two coders was used to assess the reliability of the analysis.

All participants provided informed consent to take part in this study. Patients joined the research voluntarily by invitation and were informed from the outset about the study stages and the total time required for completing questionnaires and interviews. They also signed informed consent forms for the procedure.

For the statistical analysis of the collected data, the SPSS 25 and Excel software were used.

The identification of questionnaires and interviews was done with randomly assigned codes to the patients. In all phases of the process, each participant completed the same code for subsequent processing of the material, necessary correlations, and combined commentary of the information to be carried out.

## 3. Results

### 3.1. IPAQ Results

The International Physical Activity Questionnaire (IPAQ) reflects the patients’ physical activity levels over the 7 days preceding the research period.

Among the respondents, only 1 survivor reported low PA, 6 patients reported moderate PA, and 9 patients reported high PA.

To investigate the correlation between age, body mass index (BMI), the side of the affected limb, and PA in the IPAQ, we applied the Chi-square test, leading us to the conclusion that there is no correlation between age and postoperative PA (*p* > 0.5)

Similarly, there is no correlation between BMI and postoperative PA. Ultimately, no correlation exists between the affected limb and PA after knee tumor resection and limb salvage.

### 3.2. UCLA Activity Score Test Results

Regarding the self-evaluation of survivors’ Physical Activity and Sports Activity, the patients categorize themselves on a scale from 4 to 9 in the UCLA Activity Score Test. Seven patients classified themselves in category 4 (regularly participating in mild-intensity PA); three patients classified themselves in category 5 (occasionally participating in moderate-intensity PA); two patients classified themselves in category 6 (regularly participating in moderate-intensity PA); one patient classified himself/herself in category 7 (regularly participation in sports or high-intensity PA); and three patients included themselves in category 9 (occasionally participating in impact sports).

Subsequently, we examined the correlation between age, BMI, the side of the affected limb, and PA in the UCLA Activity Score Tests, concluding that there is similarly no correlation.

### 3.3. Interviews Results

Analysis of the interview responses regarding postoperative physical activity (PA) shows that two patients engaged in one type of PA, four patients in two types, two patients in three types, six patients in four types, one patient in five types, and one patient in six types of PA.

All 16 patients reported walking as a form of physical activity, while 10 patients reported stair climbing, 10 reported shopping, 6 reported doing household chores, 4 reported gardening and playing with friends, and 1 patient reported harvesting activities as part of their physical activity (Table 2).

Furthermore, a Chi-square test was conducted to examine the relationship between PA and the complications leading to revision surgery. No significant correlation was found.

### 3.4. Sports Activity of the Patients Before Surgery/Surgeries

The semi-structured interviews also explored patients’ sports activity prior to surgery. Records indicated that 10 patients engaged in sports before surgery, while 6 had not participated in any sports activities. This suggests that a majority of the sample (10 out of 16) was quite active pre-surgery.

Patients’ choice of pre-surgery sports activities was influenced by personal enjoyment for 10 patients, encouragement from friends for 5 patients, and a perceived need for safety and self-defense for 1 patient. Among those who were active in sports, four trained more than three times per week, two participated in competitive sports, three trained 2–3 times per week, and one trained less than twice weekly.

Post-surgery, 6 out of 16 patients did not participate in any sports, while 10 continued with one sport. When asked about their interest in resuming sports post-surgery, 13 of the 16 patients expressed strong interest and anticipation. Many were eager to return to their daily routines, emphasizing the importance of maintaining good physical health post-surgery. Additionally, several noted that the positive outcome of their surgery boosted their confidence in resuming normal activities, while three patients reported that these factors had not positively influenced them.

### 3.5. Period/Time After Which the Patients Participated in Sports Activities

Concerning the period/time after which the patients participated in sports activities after surgery, four patients responded that they had never participated in sports activities. From the remaining 12 patients, 4 patients started training 1–3 months after surgery, 6 patients started training 3–6 months after surgery, and 2 patients started training after a period longer than 3 years. Finally, two patients started training but stopped due to encountering difficulties.

### 3.6. Post-Surgical Selection of Sports

Regarding post-surgical sports participation, responses indicate that 10 patients continued engaging in sports following rehabilitation. Among these, eight resumed the same sport they participated in before surgery, while two shifted to a different sport. Of the active patients, eight were involved in swimming and two in basketball; no other sports were reported.

The sample maintained similar attitudes toward sports activity (SA) post-surgery. Four patients who had not engaged in sports previously began SA, while four who were previously active discontinued. Two patients remained inactive in sports, and six continued participating with a positive outlook toward SA. Notably, all individuals who engaged in sports post-surgery participated in only one type of SA.

### 3.7. Selection Criteria of Postoperative Sports Activities

In response to the question about criteria for selecting postoperative sports activities, answers from 10 patients who engaged in sports were included. Patients could provide multiple reasons for their choice, revealing that for eight, sport selection was obligatory due to surgical considerations. Seven patients chose the sport recommended by their surgeon as part of their rehabilitation and return to normalcy. Additionally, four patients selected their sport to maintain health and strengthen their muscle system, while two chose based on personal enjoyment.

### 3.8. Duration of Postoperative Sports Activities

Among the 10 patients engaged in postoperative sports activities, 6 reported sessions lasting approximately 30 min conducted less than twice per week. Two patients trained for around 60 min, 2–3 times per week, while the remaining two patients reported training sessions of 90 min more than three times per week. Notably, no correlation was found between BMI and the duration or frequency of postoperative sports activity.

### 3.9. Role of the Patients’ Environment in Their Postoperative SA

When asked about the influence of their environment on participating in sports post-surgery, 7 patients indicated it was a personal choice, 10 mentioned their surgeon’s influence, and 7 referred to encouragement from family and friends. However, no correlation was found between environmental factors and sports activity; instead, a strong correlation was observed between personal motivation and participation in sports activities, highlighting the significant role of self-determination.

For most respondents (15 patients), their motivation to engage in sports after knee tumor resection and limb salvage was largely attributed to their surgeon’s encouragement, with only 1 patient indicating a lack of such motivation. This shows a strong association between the surgeon’s guidance and the patient’s involvement in sports activities post-surgery.

### 3.10. Type of Activity Recommended by the Medical Team

Regarding the types of activities recommended by the medical team, 15 patients reported that their surgeon initially recommended swimming as a comprehensive rehabilitation activity to strengthen muscles and gradually improve their range of motion. Additionally, 11 patients were advised to begin walking with an assistive device 2–3 days post-surgery, although they noted that walking without assistance felt manageable. Five patients were encouraged to use stairs rather than elevators, while eight received instructions to avoid certain movements, including specific gym exercises. Four patients were advised against cycling. However, two patients inquired about participating in swimming competitions, but their surgeons expressed caution due to the potential risk of continuous strain on the operated limb in competitive settings.

### 3.11. Contribution of Sports Activity to Quality of Life

Positive emotions and self-care are most frequently cited as key aspects of quality of life by 11 patients. These are followed by the energy provided to the athlete, involvement in social activities, and a sense of life purpose (mentioned by nine patients). Self-confidence was noted by eight patients, while joy, satisfaction, and mobility were referenced by seven. Fatigue was mentioned as an affirmation by four patients, and finally, financial challenges were cited by six patients, emphasizing the sample’s needs (Table 3).

## 4. Discussion

The purpose of this study was to evaluate patient attitudes and physical activity (PA) levels following knee tumor resection and distal femur megaprosthesis reconstruction, specifically examining correlations between PA levels and variables such as age, BMI, and the affected limb.

Additionally, this study assessed the impact of patients’ attitudes toward sports, the influence of family and friends, and the role of surgeons and medical teams in fostering a positive outlook on PA and SA. Finally, patients’ perspectives on the contributions of PA and SA to their post-surgical QoL were explored.

Interviews and self-evaluations indicated that all patients engaged in various forms of PA, including walking, stair climbing, shopping, and household tasks, suggesting an active lifestyle overall. The IPAQ and UCLA Activity Score Tests further supported this, with more than half of the sample demonstrating high PA levels in the IPAQ and only one patient reporting low activity. On the UCLA Activity Score, patients scored between levels 4 and 9, though interviews revealed that patients often faced mobility challenges post-surgery compared to their pre-surgical state. This difficulty was compounded by concerns about potential complications during activity, which sometimes hindered mobility. These findings align partially with other studies, which report that (a) long-term survivors of musculoskeletal tumors show significantly low levels of PA [19], (b) adult survivors treated for bone sarcoma of lower limbs during their childhood demonstrate reduced physical activity compared to the general population, which poses a potential risk to their well-being [12], (c) patients suffering from bone or soft tissue sarcomas of lower limbs constitute a unique population with high risk of physical dysfunction and chronic cardiac diseases, and (d) patients suffering from musculoskeletal tumors show severe decrease in PA with the harmful effect of prolonged inactivity [20].

The study assessed the correlation between age, BMI, affected limb, and postoperative physical activity. The Chi-square test indicated no significant correlation between these variables and physical activity levels as measured by the IPAQ. Similar findings emerged from the UCLA Activity Score Tests, aligning with the existing literature. Specifically, Lang et al. found no difference in functional outcomes or physical activity levels among patients with bone sarcoma treated with endoprosthesis reconstruction of the distal femur or proximal tibia, regardless of age or physical activity levels on the UCLA Activity Score Tests [21].

This study explored patients’ attitudes toward sports, including the types of sports they engaged in, as well as the frequency and duration of their participation. A positive attitude toward SA was prevalent, with 13 out of 16 patients expressing strong intentions for postoperative participation in SA. It is notable that this sample may be atypical, as most participants were men aged 19 to 47 for whom SA was already a lifestyle. Many respondents anticipated returning to their regular routines, highlighting the importance of maintaining good health post-surgery. Some patients also reported that the successful outcomes of their surgery boosted their confidence in resuming normal activities. Among those engaging in systematic SAs, swimming emerges as the most popular choice for postoperative activity. On the one hand, swimming might be the safest post-surgery SA option for some of the patients, as it combines muscle strengthening with minimal stress on the operated limb. On the other hand, it is primarily recommended by medical professionals for immediate exercise and rehabilitation. The sample’s SA options are in alignment with the recent bibliographic data. It is reported that the most common SAs in which patients participate are cycling or stationary biking, jogging, swimming, and specific gym exercises [15]. Also, it seems that patients begin to participate in high-impact sports, such as running, tennis, soccer, and skiing, at least three years after surgery. Regarding exercise duration and frequency, most participants reported short sessions (30 min) performed twice per week. These findings align with the recent literature, which emphasizes that the duration and frequency of exercise are key for maintaining physical health. Kisner and Colby (2003) recommend therapeutic exercise programs targeting large muscle groups (e.g., walking, running, swimming, cycling) that last over 20 min per session and are done at least 3–4 times per week [22]. However, contrary to the literature’s recommendations, most of our sample participated in exercises of short duration with low frequency per week.

The research focused on identifying a possible correlation between patients’ pre-operational and post-operative SA. Specifically, it examined whether patients’ pre-existing engagement in SA influenced an active athletic profile after the operation or whether new circumstances led to different activity choices. All patients engaged in only one SA after surgery, with no observed correlation between their SA before and after surgery. While the limited variety in postoperative SA could be linked to movement restrictions and potential weight gain, this study indicates no correlation between BMI and postoperative SA. Additionally, swimming emerged as a new activity introduced primarily for rehabilitation and muscle strengthening, gradually becoming a consistent lifestyle choice. No conclusions were made regarding the intensity level of SA, as this would require specific laboratory measurements.

Findings, also suggest that a successful initial surgery positively supports participation in SA, although the small sample size and patients’ consistent view of their surgery as successful limited further exploration. Unlike existing studies, there was no notable decline in either physical or sports activity levels [23].

No significant correlation was found between postoperative SA and these external factors, such as family and social environment on the patients’ participation in SAs. However, a strong link was observed between SA and personal motivation. Many patients cited “it was my choice”, indicating a high degree of self-perception as a motivator.

The influence of surgeons and physiotherapists on SA participation revealed a strong correlation. This aligns with existing research highlighting the role of surgeons and medical staff in patients undergoing distal femur resection with endoprosthesis reconstruction for bone sarcoma treatment [24,25,26]. According to Hobusch et al., patient rehabilitation following distal femur resection with endoprosthesis reconstruction for bone sarcoma is most effective in specialized centers, where continuous contact with surgeons and medical teams optimizes mobility [19]. Patient responses confirmed their adherence to their surgeons’ recommendations, often including immediate mobilization through swimming and walking to facilitate a quicker return to daily routines.

This study also addressed whether the patients who have undergone limb-salvage surgery for bone sarcoma treatment consider sports an element of their QoL. Patients acknowledged the role of environmental access and, notably, the financial commitment required for consistent rehabilitation as crucial factors for maintaining a good QoL. The high cost of ongoing support may be the primary factor impacting QoL for the sample, consistent with findings in other studies [10,27].

### Limitations

This study has several limitations. Firstly, the sample size was relatively small, which may affect the generalizability of the findings. However, this study constitutes a good basis for further investigation into this matter. Additionally, the geographical spread of patients across the country, along with the challenges they encountered in traveling to meet the researcher in person, limited the collection of further data that could have enhanced the study. Moreover, there are only a few orthopedic surgeons and specialized teams for this type of surgery, and not all doctors encourage their patients to participate in research. Cancer is often linked to metastases, meaning that even when surgeries are successful, long-term survival is not guaranteed, particularly in the cases of metastases. Patients also tend to be reluctant to share their experiences, often feeling self-conscious about body changes or life adjustments following surgery. In Greek culture, openly sharing personal challenges with researchers or the public is uncommon, making it difficult to obtain patient consent for research participation.

## Figures and Tables

**Table 1:** Sample characteristics of the 16 patients with distal femur resection and limb salvage, who participated in this study.

Ν	Gender	Age	FU	Diagnosis	Site (R = Right, L = Left)	Chemotherapy	RadiationTherapy	Complications	Reoperation
AΚ49	M	33	23	Osteosarcoma	L	YES	NO	Loosening	YES
AΚ31	M	32	16	Osteosarcoma	L	YES	NO	NO	
AΚ54	M	45	6	Chondrosarcoma	L	NO	NO	Breakage	YES
AΚ26	M	47	8	Chondrosarcoma	L	NO	NO	NO	
AΚ53	M	46	18	Pleomorphic Sarcoma	L	YES	NO	NO	
ΓΚ46	F	41	24	Osteosarcoma	L	YES	NO	NO	
ΓΚ32	F	32	5	Osteosarcoma	L	YES	NO	Loosening	YES
AΚ37	M	40	9	Chondrosarcoma	R	NO	NO	Breakage	YES
ΓΚ19	F	26	4	Ewing Sarcoma	R	YES	NO	NO	
AΚ64	M	31	15	Osteosarcoma	L	YES	NO	NO	
AΚ44	M	31	5	Osteosarcoma	L	YES	NO	Loosening	YES
ΓΚ52	F	19	3	Osteosarcoma	L	YES	NO	Infection	YES
AΚ24	M	24	7	Osteosarcoma	L	YES	NO	Loosening	YES
AΚ50	M	30	13	Ewing Sarcoma	R	YES	NO	Loosening	YES
AΚ30	M	25	10	Chondrosarcoma	R	NO	NO	NO	
AΚ20	M	29	3	Osteosarcoma	L	YES	NO	NO	

**Table 2:** Distribution of specific physical activities of 16 patients with distal femur resection and limb salvage.

Postoperative Physical Activity—Frequencies		
Physical Activity	Number	Percentage
walking	16	31.4%
stair climbing	10	19.6%
housework	6	11.8%
gardening	4	7.8%
shopping	10	19.6%
playing with friends	4	7.8%
heavy labor/agricultural work	1	2.0%
Total	51	100.0%

**Table 3:** Contribution of sports activity to quality of life, as mentioned by the patients.

Quality of Life	Number	Percentage
Energy	9	10.2%
Fatigue to prove that “I can do it”	4	4.5%
Positive feelings	11	12.5%
Self-Confidence	8	9.1%
Joy	7	8.0%
Satisfaction	7	8.0%
Motivation	7	8.0%
Self service health care	11	12.5%
Participation in social activities	9	10.2%
Meaning to the life	9	10.2%
Financial problems-lack of infrastructures-Difficulties in accessibility	6	6.8%
TOTAL	88	100%

## Data Availability

All data supporting the results are available upon request, without prejudice to privacy or ethical restrictions.
